# An Overarching Conceptual Framework for Menstrual Health in Sport Research: Theory, Causality and Validation

**DOI:** 10.1007/s40279-026-02411-w

**Published:** 2026-03-23

**Authors:** Chelsea Oester, Dean Norris, Ric Lovell, Charles Pedlar, Georgie Bruinvels, Judd T. Kalkhoven

**Affiliations:** 1 School of Health Sciences, Western Sydney University, Campbelltown Campus, Narellan Rd & Gilchrist Dr, Campbelltown, Sydney, NSW 2560 Australia; 2 Faculty of Science, Medicine and Health, University of Wollongong, Wollongong, NSW Australia; 3 Faculty of Sport, Health and Applied Science, St Mary’s University, London, UK; 4 Orreco Business Innovation Unit, National University of Ireland, Galway, Ireland; 5 Institute of Sport, Exercise and Health, University College of London, London, UK

## Abstract

Menstrual health in sport is a complex domain shaped by biological, psychological and contextual factors. Central to these interactions is the menstrual cycle, a fundamental physiological process that affects female athletes across multiple dimensions. While research in this area is growing, it often lacks a unifying sport-specific framework to guide theory development, data interpretation and practical application. This article addresses this gap by proposing an overarching conceptual framework (i.e. nomological network) to integrate diverse constructs related to menstrual health in sport to support a more coherent theory-driven approach across both laboratory and field settings. The framework brings together key elements, with the construct of *menstrual-related effects* representing the primary mechanisms through which menstrual-related phenomena are theorised to causally influence sport-related outcomes such as performance, health, participation and behaviour. When constructs are linked to outcomes through clearly identified mechanistic pathways, it enhances the biological and theoretical plausibility of any proposed relationship, reinforces its justification within a broader system of theory, and strengthens the evidential basis for validation. However, while useful for organising constructs, shaping research questions and hypotheses, and stimulating theory-driven inquiry, the proposed framework is largely informal and therefore offers primarily heuristic value. It is insufficient on its own for formalised empirical testing. For this reason, the adoption of causal directed acyclic graphs is advocated for investigating specific research questions through robust statistical analysis, causal modelling and validation. Directed acyclic graphs are mathematical models that explicitly encode variables, hypothesised causal pathways, mechanisms and confounders in a formal causal structure that enables systematic and testable estimation of causal effects, including from observational data. This approach enhances transparency and interpretability, facilitates refinement of model specifications and supports more rigorous validation processes. Ultimately, the integration of a heuristic conceptual framework with the formal methodology of causal directed acyclic graphs provides both a structured and theory-driven foundation for organising knowledge and the formal modelling approach required to address specific research questions and strengthen empirical inquiry in menstrual health in sport research.

## Key Points

**Table:** 

This article proposes a nomological network as an overarching heuristic framework for menstrual health in sport research, organising key constructs and their inter-relationships to guide theory-driven research and mechanistic reasoning.
Within the framework, menstrual-related effects serve as the primary mediator through which biological, interventional and wider contextual variables are hypothesised to influence sport-related outcomes, such as performance, health, participation and behaviour.
For answering specific research questions through robust statistical analysis, causal modelling and validation, causal directed acyclic graphs are recommended as they explicitly represent assumptions, mechanisms and confounders within a formalised and testable mathematical structure.
While causal directed acyclic graphs provide a general framework for causal reasoning applicable across study designs, including randomised, prospective and observational research, in the context of menstrual health and female athlete research, this approach may be particularly beneficial because of the ethical, biological and practical constraints that often limit the feasibility of randomised controlled trials, thereby enabling explicit specification and testing of causal assumptions using observational data.

## Introduction

In recent years, there has been a surge in public interest and research focused on the menstrual cycle (Table 1) and menstrual health, particularly within the realm of sports. This trend can be partly attributed to the growing advocacy for recognising healthy menstrual cycle functioning as a vital sign of overall female health [1], coupled with increased media attention on the connection between menstrual cycle-informed training design and athletic success, as well as the potential negative impact of menstrual symptoms on performance [2]. Current theories in this area primarily focus on the effects of the main female reproductive hormones, oestrogen and progesterone (Table 1), whereby fluctuations of these hormones are widely hypothesised to influence a range of psycho-physiological systems [3]. In turn, these hormonal fluctuations, and the effects that they induce on the human body, potentially impact a variety of sports-related outcomes of interest, such as athletic performance [4–6] and injury risk [7, 8]. However, menstrual health in sport is a broad and multi-faceted research area that is not simply reducible to hormones and their potential effects on athletic performance and injury. Rather, it encompasses several important research domains. These include but are not limited to the effects of menstrual health and management on sports participation [9, 10]; the socioeconomic and cultural environments relating to menstrual health and its effective management in athletes [11–14]; social dynamics within sporting environments [15]; and the psychological and physical well-being of athletes [16]. For the purposes of this article, and adapted from the definition of menstrual health developed by Hennegan et al. [17], and used by the World Health Organization [18], menstrual health in sport is defined as follows:

“*a state of complete physical, mental, and social well-being acknowledging the unique challenges and considerations associated with the menstrual cycle and sport, encompassing all treatments, interventions and exposures acting on the menstrual cycle and its related effects, with the goal of attaining athletic potential, supporting athlete well-being, and fostering a holistic health-centred sporting environment*”

**Table 1:** Relevant nomenclature

Operational definitions	
Abnormal uterine bleeding (AUB)	Implicitly, non-gestational and in the reproductive years. Any alteration in the normal frequency, regularity, duration or volume of menstrual bleeding (including heavy menstrual bleeding) as well as intermenstrual bleeding and unscheduled bleeding with pharmaceutical agents designed to suppress menstrual function [30]
AUB - Endometrial (former luteal phase defect)	Abnormal bleeding due to inadequate levels of progesterone secretion [31]. Historically termed “luteal phase defect”, this concept is debated in reproductive medicine, where no consensus diagnostic test or treatment has been validated, and the luteal phase defect is not currently recognised as a distinct clinical entity [32]
AUB - frequent menstruation (former polymenorrhoea)	Menstrual cycle duration of less than 24 days [30]
AUB - infrequent menstruation (former oligomenorrhoea)	Defined as a menstrual cycle length of more than 38 days [30]. Infrequent menstruation can arise from multiple aetiologies, each with distinct hormonal profiles, and can indicate potential disturbances in reproductive function (e.g. anovulatory cycle) [40]
Anovulation (AUB - Ovulatory dysfunction)	Failure to ovulate [30], typically presenting as a bleed without an egg release and without the expected rise in progesterone levels in the second half of the cycle [22]. In applied sport science, anovulation is usually inferred when a single mid-luteal progesterone measurement falls below a specific threshold, whereas reproductive medicine cautions that no single-time measure is sufficient because of pulsatile secretion. Instead, cycle length, serial progesterone profiles or combined criteria (e.g. short luteal phase plus low progesterone) should be considered [32]
Causal mediation analysis	Causal mediation analyses use statistical methods to examine how an independent variable influences an outcome through one or more mediator variables, distinguishing the effects into direct and indirect (mediated) pathways [24]
Corpus luteum	A temporary endocrine structure in the ovary that forms from the remnants of the ruptured follicle after ovulation. It secretes hormones, primarily progesterone and some oestrogen, to prepare and maintain the uterine lining for a potential pregnancy. If fertilisation does not occur, the corpus luteum degenerates, leading to menstruation [33]
d-separation	d-separation (directional separation) is a criterion used in causal graphs (DAGs) to determine whether a set of variables blocks all paths, and thus all potential sources of association, between two other variables. If a set of variables d-separates two nodes, then those nodes are conditionally independent given that set [27]
Endometrial cycle	The series of changes that occur in the lining of the uterus (endometrium) during a menstrual cycle, preparing it for the potential implantation of a fertilised egg. It includes the menstrual phase (shedding of the lining), the proliferative phase (regeneration and thickening of the lining) and the secretory phase (further preparation for possible pregnancy) [34]
Endometriosis	A disease characterised by the presence of endometrium-like epithelium and/or stroma outside the endometrium and myometrium, usually with an associated inflammatory process [35]. Common subtypes include peritoneal/superficial endometriosis (lesions involving the peritoneal surface), ovarian endometrioma (cystic ovarian lesions), deep endometriosis (lesions infiltrating abdominal structures) and bowel endometriosis (lesions within the bowel wall) [35]
Endometrium	The inner lining of the uterus that thickens and becomes more vascular throughout the menstrual cycle, preparing to support a potential pregnancy. If fertilisation does not occur, it is shed during menstruation [34]
Eumenorrheic	Characterised by a menstrual cycle length between 21 and 35 days, having at least nine menstruations per year, no use of hormonal contraceptives in the previous 3 months, evidence of an LH surge indicating potential ovulation, and the expected hormonal profile (mid-cycle oestradiol rise followed by an oestradiol and progesterone increase mid-luteal) [22]. For individuals who have recently discontinued hormonal contraceptive use, the criterion of nine menstruations per year may not be applicable
Exposure	The condition of being subjected to something (such as a stressor, agent or factor) that may influence health or performance [24]
Follicle-stimulating hormone (FSH)	Hormone released by the pituitary gland in response to the release of gonadotropin-releasing hormone from the hypothalamus. FSH promotes the growth and maturation of ovarian follicles, which are structures that contain eggs [36]
Functional hypothalamic amenorrhoea (FHA)	A form of hypogonadotropic hypogonadism caused by disrupted pulsatile GnRH release from the hypothalamus. Hypothalamic-pituitary disturbances encompass a wide range, from the complete absence of LH pulsatility to a seemingly normal secretion pattern and higher mean frequency of LH pulses [37]. Clinically, FHA is a common cause of secondary amenorrhoea, typically defined as ≥ 3 months without menstruation [37]
Gonadotropin-releasing hormone (GnRH)	The hormone released by the hypothalamus into the bloodstream, which triggers the pituitary glands to release LH and FSH [36]
Heavy menstrual bleeding	Excessive menstrual blood loss that interferes with a woman’s physical, social, emotional and/or material quality of life [30]
Hyperprolactinemia	Elevated levels of prolactin in the blood, which negatively modulate the secretion of LH and FSH. An excess of prolactin is associated with a short luteal phase (< 10 days) and infertility [38]
Identification	Identification (in causal inference) is the condition that, given stated causal assumptions, a target causal estimand e.g. P(Y ∣ do(X = x)), is uniquely determined (i.e. expressible by a formula) from the observed data distribution [26, 27, 29]
Hormonal contraceptive methods	Prevent pregnancy through one or more mechanisms, including suppression of ovulation, thickening of cervical mucus and endometrial thinning. These methods are effective and reversible and include oral contraceptives (the pill), hormonal intrauterine devices, vaginal rings, implants and injectables [39]
Hypothalamic-pituitary-ovarian (HPO) axis	Comprises the hypothalamus, pituitary gland and ovaries and regulates the female reproductive system through complex neuroendocrine feedback loops [36]
Luteinising hormone (LH)	Hormone released by the pituitary gland in response to the release of GnRH from the hypothalamus. LH triggers ovulation and stimulates the production of oestrogen and progesterone from the ovaries [36]
Mediator	A variable that explains the mechanism through which two variables are related, often indicating how or why a particular effect or relationship occurs [24]
Menstrual cycle	The duration in days from the first day of one menstrual period to the first day of the next [30]. In reproductive-age women, the typical cycle length ranges from 21 to 35 days, although substantial variability occurs both between and within individuals [22]
Naturally (regularly) menstruating	Characterised by a menstrual cycle length between 21 and 35 days, having at least nine menstruations per year, no use of hormonal contraceptives and defined by bleeding pattern alone without confirmation of ovulation or the expected hormonal profile [22]
Nomological network	A nomological network is a conceptual framework that defines a construct by specifying its relationships with other observable and theoretical variables. It is used to guide validation by showing how a construct fits within a broader system of expected associations [21]
Non-hormonal contraceptive methods	Hormone-free methods preventing pregnancy, such as copper intrauterine device, condoms and vasectomy or female sterilisation [39]
Oestradiol	A hormone that is produced in the ovaries and is the most abundant and potent form of oestrogen in reproductive aged women. It plays a major role in the regulation of the menstrual cycle and in the development of secondary sexual characteristics [36]
Oestriol	A hormone that is primarily produced during pregnancy by the placenta. It is the least potent form of oestrogen, meaning it has less affinity for oestrogen receptors than oestradiol and therefore may have a weaker effect on the body compared with other forms of oestrogen [36]
Oestrogens	A group of steroid hormones that promote the development and maintenance of female characteristics in the body, playing a key role in the regulation of the menstrual cycle [36]
Oestrone	A hormone that is the weaker form of oestrogen relative to oestradiol and produced mainly in fat tissue. It becomes the predominant type of oestrogen after menopause when the production of ovarian oestradiol decreases [36]
Outcome	The result or effect of an action, situation or event, often used to measure the effectiveness of a treatment, intervention or exposure [24]
Ovarian cycle	The series of events that occur in the ovaries during a menstrual cycle, involving the development and maturation of ovarian follicles, the release of a mature egg during ovulation and the formation of the corpus luteum, which produces hormones to prepare for potential pregnancy [34]
Ovulation	The release of an oocyte (egg) from an ovarian follicle [30]. Serial follicular scanning via transvaginal ultrasonography is the gold standard method to confirm ovulation [41]
Premenstrual dysphoric disorder (PMDD)	A severe form of premenstrual syndrome marked by intense mood and physical symptoms occuring before menstruation and subsiding shortly after menstruation. The diagnosis requires at least five symptoms, including mood issues such as irritability or depression, which significantly impair daily functioning. These symptoms must be confirmed over two cycles and cannot solely result from another disorder [42]
Premenstrual syndrome (PMS)	A wide range of physical, psychological and behavioural symptoms that occur for several days before menstruation and subside shortly after menstruation [43]
Polycystic ovary syndrome (PCOS)	A heterogenous hormonal disorder diagnosed in women presenting with at least two of the three following characteristics: hyperandrogenism (hirsutism and/or hyperandrogenaemia), chronic anovulation and polycystic ovarian morphology [44]
Primary amenorrhoea	Failure of onset of menstruation by the age of 15 years [30] or 14 years when no secondary sexual characteristics are present [22]
Primary dysmenorrhoea	Menstrual bleeding accompanied by significant pain (usually cramps) without an underlying medical condition [45]
Primary ovarian insufficiency	Where ovaries stop functioning normally before the age of 40 years, which can result in amenorrhoea, infertility and levels of FSH in the post-menopausal range [46]
Progesterone	A steroid hormone released by the corpus luteum in the ovaries after ovulation that regulates the menstrual cycle and supports pregnancy. During pregnancy, the placenta also produces progesterone [36]
Relative energy deficiency in sport (REDs)	Impaired physiological and/or psychological functioning experienced by female and male athletes that is caused by exposure to problematic (prolonged and/or severe) low energy availability. The detrimental outcomes include, but are not limited to, decreases in energy metabolism, reproductive function, musculoskeletal health, immunity, glycogen synthesis and cardiovascular and haematological health, which can all individually and synergistically lead to impaired well-being, increased injury risk and decreased sports performance [47]
Secondary amenorrhoea	Absence of menstrual periods for more than 3 months in non-pregnant women with previous regular cycles, or over 6 months in those with previously irregular cycles [48]. Secondary amenorrhoea can result from multiple aetiologies, each associated with different hormonal profiles. For instance, FHA is characterised by low oestrogen, whereas PCOS can present with normal oestrogen levels [48]
Secondary dysmenorrhoea	Painful menstruation accompanied by an underlying medical condition or disorder (e.g. endometriosis) [45]
Sensitivity analysis	In the context of causal inference, sensitivity analysis refers to methods used to assess how robust causal conclusions are to potential violations of key assumptions, such as unmeasured confounding, model misspecification or measurement error. It helps determine whether the estimated causal effect would change substantially if these assumptions were not fully met [27]
Withdrawal bleeding	Bleeding caused by a drop in exogenous hormones when using certain types of hormonal contraceptives [36]

*AUB* abnormal uterine bleeding, *DAGs* directed acyclic graphs, *FHA* functional hypothalamic amenorrhoea, *FSH* follicle-stimulating hormone, *GnRH* gonadotropin-releasing hormone, *LH* luteinising hormone, *PCOS* polycystic ovary syndrome

Within the menstrual health literature, various models and frameworks have been presented [17, 19, 20]. However, these are not designed for a sporting context, instead addressing the general population of women with a primary focus on overall health and well-being. As such, they do not account for the unique challenges and circumstances faced by athletes, who must navigate menstrual health within a performance-oriented sporting environment. To support researchers in understanding the broad and complex nature of menstrual health in sport, the development of an overarching conceptual framework is warranted. The framework presented within this article draws upon the principles of a nomological network (Table 1), as outlined by Cronbach and Meehl [21], to build upon previous methodological recommendations by Elliott-Sale et al. [22]. It organises key concepts and relationships to help guide validation processes and strengthen causal inferences, the importance of which has been increasingly emphasised across the sports science and medicine literature [23–25]. In this way, the framework serves as a high-level conceptual map that breaks down this complex area into relevant theories, assumptions and proposed causal links [24], with the aim of guiding study design, supporting validation, and encouraging the synthesis of findings from both laboratory and field-based studies.

However, while this high-level framework provides a useful organising structure, its broad scope and generality limit its utility for direct empirical testing and precise validation. To overcome this, the adoption of causal directed acyclic graphs (DAGs) is advocated for answering specific research questions through robust statistical analysis, causal modelling and validation. Unlike overarching conceptual frameworks, which are largely informal and primarily offer heuristic value, causal DAGs are formal mathematical models that provide explicit and testable representations of hypothesised causal pathways, mediating mechanisms, and potential confounders. Their use is supported by a comprehensive mathematical foundation, including graphical criteria (e.g. d-separation; Table 1) and rigorous theorems such as the do-calculus, which underpin their capacity for robust causal inference [26–28]. They enable researchers to evaluate whether relationships between measures behave as theoretically expected within a structured system and to determine whether a given set of variables is conditionally independent, as theorised, given a specified adjustment set [26–28]. By making assumptions explicit and strengthening model transparency, DAGs support more precise study designs, improve model specification and enhance the interpretability of findings [26–28]. While applicable to experimental and prospective designs, causal DAGs can also leverage observational data to support causal inference and validation when randomised controlled trials (RCTs) are infeasible [26–29].

This article follows a clear and logical structure, thoughtfully designed to accommodate readers with varying levels of expertise in menstrual health in sport research. It begins with guidance on interpreting the framework from a causal inference perspective, followed by the presentation of the overarching framework in Sect. 3. Sections 4 through 7 provide concise overviews of the framework’s key components—broad intuitive constructs that are not elaborated in detail here, but are linked to more comprehensive sources for those seeking deeper engagement. Section 8 then shifts to practical application, illustrating how the framework can guide research processes and support more rigorous validation efforts through the use of causal DAGs.

**Note:** As this article is intended for a broad audience, readers already familiar with menstrual health in sport research, and elements of the framework presented, may choose to engage selectively with introductory sections, while the later section on causal DAGs may be more relevant to their interests.

Finally, the framework presented here should be understood as a flexible overarching structure rather than a fixed model. It organises current knowledge to aid in research question development, hypothesis formation and gaining a general understanding of the field, but may lack specificity for addressing particular research problems. Accordingly, other researchers are encouraged to adapt, refine or expand it to suit their own research questions, contexts or populations. In this sense, the framework is designed to remain open to revision, expansion or replacement should it not serve its purpose in effectively conceptualising specific problems, reflecting the evolving nature of science while catering to the diverse needs of menstrual health research. Looking forward, as the field progresses, it is envisaged that formalised causal DAGs will be developed and tested to provide a more robust means of examining specific research questions and to offer a stronger foundation for causal interpretations of findings.

## Temporal and Hierarchical Dynamics of Variables in Causal Inference: Implications for Interpretation of the Framework

Understanding cause–effect relationships is a fundamental goal of science [49]. Causal knowledge facilitates the development and implementation of targeted interventions to influence specific outcomes of interest through causal effects [24, 26–28]. Given the critical role of causality in scientific inquiry, it is essential to clarify the interpretation of causality used within this framework prior to presenting it. Notably, various perspectives on causation exist, such as dynamical systems [50, 51] and potential outcomes [52]. This article adopts the causal inference perspective, specifically the structural causal model approach, for understanding causality [26–28].

**Fig. 1:**
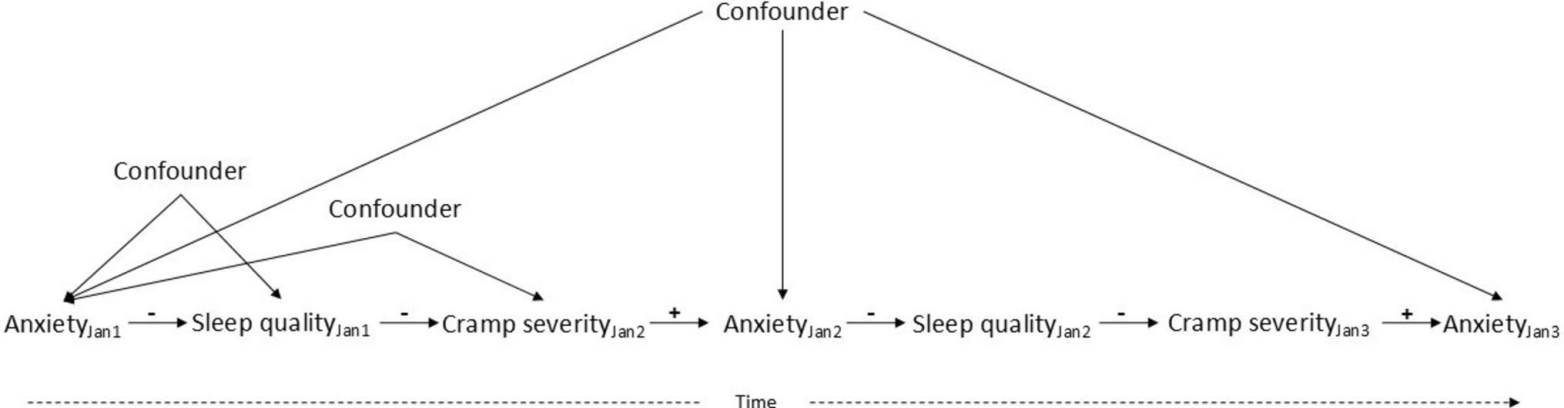
Example of a causal directed acyclic graph illustrating how a single construct, such as anxiety, can be represented as distinct variables at different timepoints (Anxiety_Jan1_, Anxiety_Jan2_, Anxiety_Jan3_). This temporal distinction allows the same construct to function as an exposure (Anxiety_Jan1_), mediator (Anxiety_Jan2_), and outcome (AnxietyJ_an3_) within a single framework. The *arrows* in the directed acyclic graph represent hypothesised direct causal effects, meaning that changes in one variable are assumed to causally influence another. The ( +) symbols indicate direct positive causal effects, where increases in one variable lead to increases in the variable it affects (e.g. greater cramp severity causes greater anxiety). The ( −) symbols indicate direct negative causal effects, where increases in one variable lead to decreases in the affected variable (e.g. higher anxiety leads to reduced sleep quality). A confounder is a common cause of a selected treatment and outcome that, if not appropriately controlled for, can bias the estimated relationship between them. Note: this figure is intended solely as an illustrative example of how causal logic and temporal dynamics can be represented in a directed acyclic graph. It should not be interpreted as a validated causal model or as evidence of established causal effects. While some associations consistent with these links have been reported in the literature, formal causal inference methods are required to establish valid causal effects

Within causal inference, the timing of data collection and the manner in which variables are measured play a critical role in how variables are represented and interpreted within a causal model. A construct that appears to be a single variable may, when measured at different timepoints, correspond to distinct variables within a DAG. For example, the oestradiol level measured on different days of the menstrual cycle represents temporally distinct variables rather than repeated observations of a single causal entity. These temporally indexed variables are causally linked and may assume different roles within the research process, functioning as an exposure at one timepoint, a mediator at another and an outcome at a later stage (Fig. 1; Table 1).

To illustrate the logical structure and representation of variable relationships over time within a causal framework, an example of a causal DAG is presented in Fig. 1. In this example, anxiety is modelled across three timepoints (Anxiety_Jan1_, Anxiety_Jan2_, Anxiety_Jan3_), demonstrating how the same construct can function as an exposure, mediator or outcome depending on when it is measured. The arrows represent hypothesised causal relationships and show how temporal dependencies can be encoded (e.g. Anxiety_Jan1_ influencing sleep, sleep influencing cramp severity, cramp severity influencing subsequent Anxiety_Jan2_). Although some associations consistent with these links have been reported in the literature [53–55], this example should not be interpreted as a validated causal model or as evidence of established causal effects. A rigorous evaluation of such relationships requires a carefully specified causal model and the application of appropriate statistical methods. The example is therefore strictly intended as a didactic demonstration of how variables may shift roles within a causal system, rather than as a proposal of empirically verified causal relationships.

Importantly, the same principles extend beyond temporal indexing to differences in levels of abstraction, which have direct implications for causal representation. As a general principle, constructs measured at different biological or contextual levels are not interchangeable and should not be treated as causal peers within the same causal DAG. As one illustrative example, systemic hormone levels and menstrual cycle phase operate at different levels of abstraction: hormone concentrations represent biological state variables with mechanistic causal relevance, whereas menstrual phases are higher-level descriptive classifications defined by underlying hormonal trajectories. In such cases, menstrual phase does not constitute an independent causal variable once hormonal states are specified, and including both within the same causal DAG would amount to modelling a variable alongside its own abstraction.

These considerations directly inform how variables are organised and interpreted within the proposed framework. While the framework organises and highlights key concepts within a structured schematic, what may initially appear to be a single variable can, depending on when, how and at which level of abstraction it is measured, assume different roles within the framework, such as functioning as a contextual factor, a symptom or an outcome of interest. The more specific implications of this distinction for causal modelling and interpretation in menstrual health research are discussed in greater detail in Sect. 8.

## An Overarching Conceptual Framework for Menstrual Health in Sport Research

**Fig. 2:**
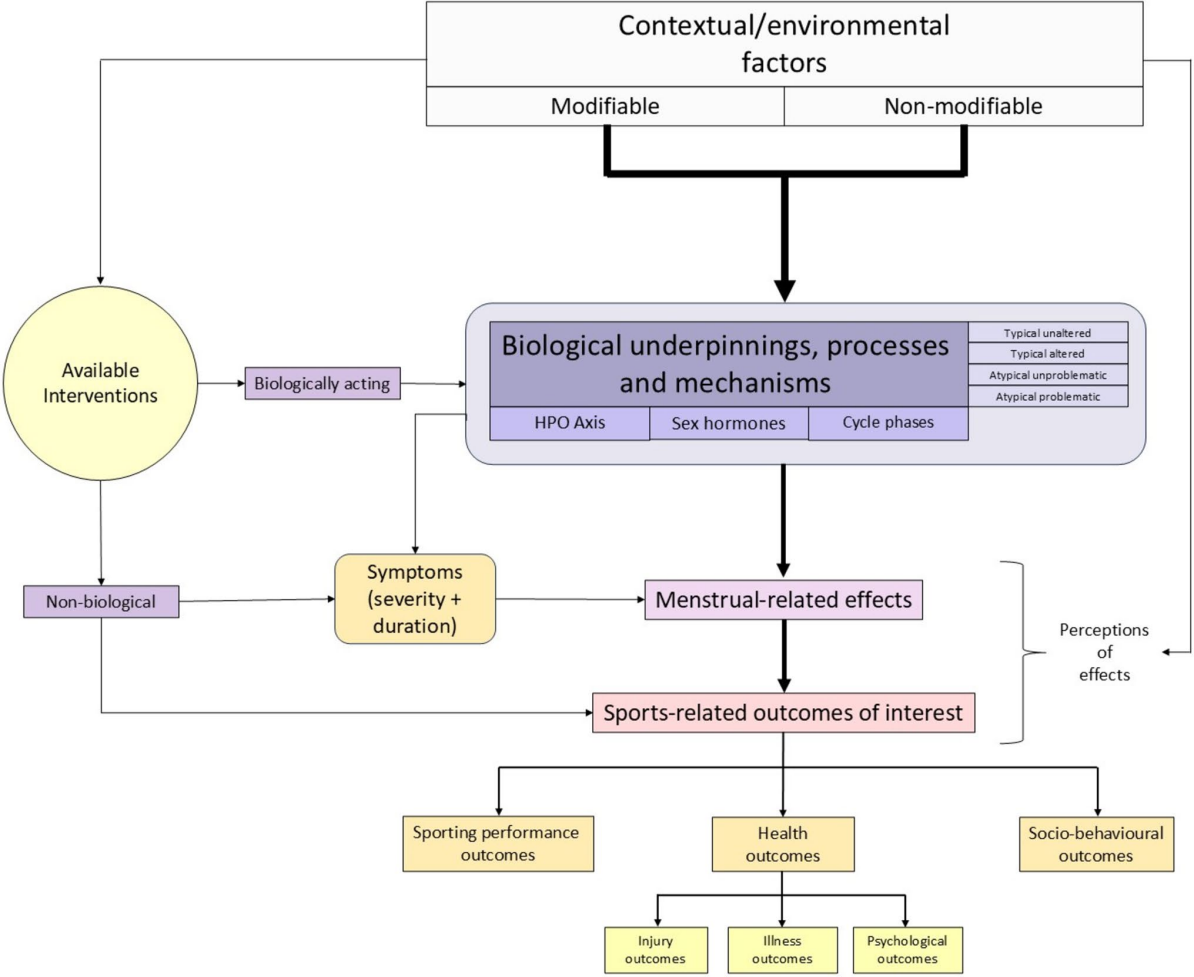
A high-level conceptual framework (i.e. nomological network) for menstrual health in sport research. The figure illustrates the key constructs and their proposed interrelationships, with *directional arrows* representing hypothesised causal pathways. The framework integrates contextual and environmental factors, biological processes and mechanisms (e.g. the hypothalamic-pituitary-ovarian axis, hormonal fluctuations, menstrual phases), and their potential influence on sport-related outcomes such as performance, health and sociobehavioural factors. These influences are theorised to operate primarily through menstrual-related effects, which function as the central mediating mechanisms. The framework also incorporates menstrual symptoms as well as biologically and non-biologically acting interventions. As a high-level and largely heuristic structure, it is intended to provide an organising scaffold for generating hypotheses, informing research design and supporting validation efforts, thereby advancing understanding of menstrual health in sport. *HPO* hypothalamic-pituitary-ovarian

Figure 2 provides a high-level conceptual framework (i.e. nomological network) for menstrual health in sport research.

## Contextual/Environmental Factors

Within the proposed framework, contextual/environmental factors represent a broad construct that includes a myriad of variables that may influence relevant sports-related outcomes of interest by acting on either the menstrual cycle and its symptoms, or the accessibility, adoption or utilisation of various menstrual-related interventions. To help organise these, contextual/environmental factors are categorised within the framework as either modifiable or non-modifiable. Given the extensive range of variables that may fit within these domains, only some of the more prominent variables identified in the literature are highlighted as examples.

Modifiable factors influencing both the menstrual cycle and its management, and that are associated with short- and long-term sports-related outcomes of interest, typically encompass lifestyle-related variables. For example, disrupted sleep patterns can affect circadian rhythms, impairing the hypothalamic-pituitary-ovarian (HPO) axis (Table 1) and altering the release of reproductive hormones, which may lead to menstrual irregularities [56, 57]. Additional lifestyle factors include low energy availability [58] and exercise habits [59]. Cultural, religious and socioeconomic considerations [60], along with menstrual health literacy [61], also represent modifiable influences that have been explored within the literature. Examples of non-modifiable factors include age [62], genetics [63], past experiences [64] and underlying reproductive health conditions such as polycystic ovary syndrome (PCOS) [Table 1] [65].

## Biological Underpinnings, Processes and Mechanisms of the Menstrual Cycle

While menstrual health is multi-faceted, the menstrual cycle itself is a biological phenomenon. Therefore, the biological underpinnings, processes and mechanisms of the menstrual cycle constitute a fundamental element of the proposed framework. As these aspects have been comprehensively detailed elsewhere [66–68], the present article provides only a concise overview of those components and processes that are considered central to the menstrual cycle and its functions. This includes the HPO axis, the primary female sex hormones (oestrogen and progesterone), the distinct phases of the menstrual cycle and approaches to tracking them, and alterations in reproductive hormonal profiles across the life span.

### Hypothalamic-Pituitary-Ovarian (HPO) Axis

**Fig. 3:**
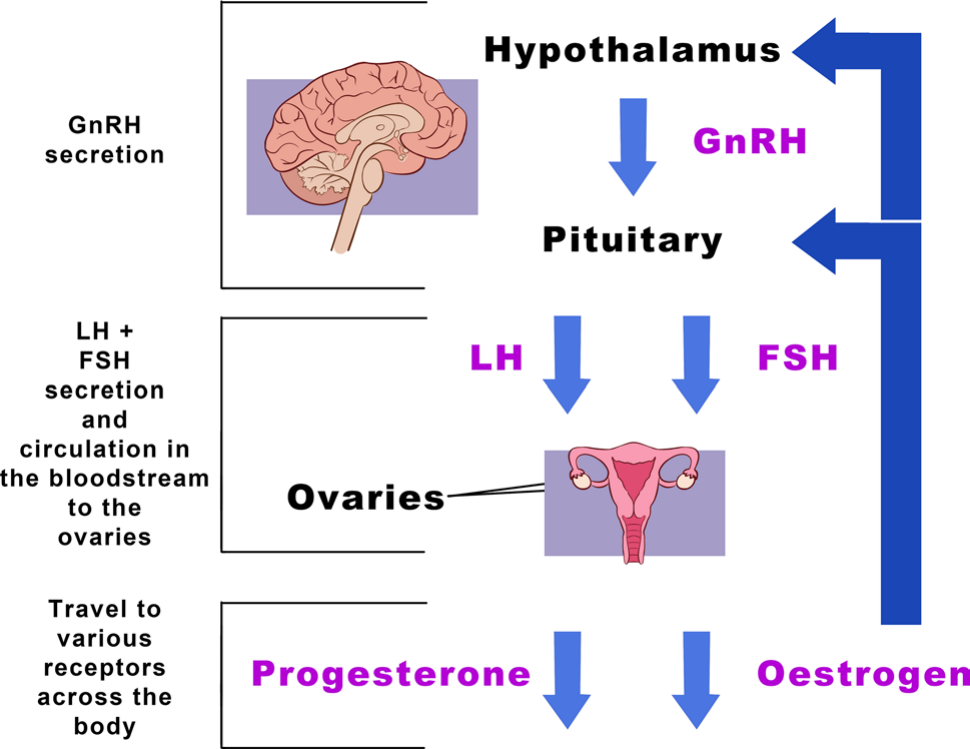
The hypothalamic-pituitary-ovarian axis. The hypothalamus, located at the base of the brain, is tasked with releasing gonadotropin-releasing hormone (GnRH), which then travels to the pituitary gland, also located in the base of the brain, where it stimulates the release of luteinising hormone (LH) and follicle-stimulating hormone (FSH) [69, 70]. These two hormones subsequently circulate to the ovaries, located on either side of the uterus, where they bind to specific hormone receptors, triggering the production and release of oestrogen (specifically oestradiol) and progesterone [71]. The hypothalamic-pituitary-ovarian axis is regulated by multiple feedback loops. During the follicular phase, rising oestradiol secreted by developing ovarian follicles exerts negative feedback on the hypothalamus, downregulating GnRH secretion. At the peak of follicle maturation, high oestradiol levels trigger a positive feedback response that initiates the LH surge required for ovulation. In the post-ovulatory period, oestradiol and progesterone secreted by the corpus luteum exert negative feedback, reducing the GnRH pulse frequency. These feedback mechanisms are essential for regulating pulsatile GnRH release and maintaining the cyclical pattern of reproductive hormone secretion [34]

The intricacies of the menstrual cycle are primarily regulated by the HPO axis, a critical endocrine system consisting of the hypothalamus, the pituitary gland and the ovaries [36] (Fig. 3). Each of these components of the HPO axis has a distinct role, yet their processes are intricately connected, as illustrated in Fig. 3.

The HPO axis is a sensitive system, and even subtle disruptions to its feedback mechanisms from contextual and environmental factors, such as low energy availability [72], excessive exercise [73], psychological stress [74], sleep disturbance [57], illness [75], or other physiological and environmental stressors [74], can significantly alter menstrual cycle characteristics. These disruptions may, for example, affect gonadotropin-releasing hormone, which in turn impairs the release of luteinising hormone and follicle-stimulating hormone, as well as the subsequent secretion of oestrogen and progesterone, ultimately hindering ovulation (Table 1) [76].

### Female Sex Hormones: Oestrogen and Progesterone

Oestrogen and progesterone are essential for regulating the female reproductive system, driving sexual development, controlling the menstrual cycle and supporting pregnancy. Oestrogen refers to a group of steroid hormones that includes oestradiol, oestrone and oestriol (Table 1). Oestradiol, the most potent and prevalent form, is essential for the development of secondary female sexual characteristics, such as breast development, and for regulating the menstrual cycle by promoting the growth of the uterine lining (endometrium) [Table 1] in preparation for a potential pregnancy [36]. Beyond reproductive functions, oestrogens play a role in various physiological processes, including supporting bone health [77], promoting cardiovascular function [78], maintaining redox homeostasis to protect cells and tissues from oxidative damage [79], and regulating mood and cognitive function [80].

Progesterone is primarily produced by the corpus luteum (Table 1) in the ovaries after ovulation, and by the placenta during pregnancy [81]. Its main functions include preparing the endometrium for the implantation of a fertilised egg and maintaining pregnancy by preventing the shedding of the uterine lining [82]. Additionally, progesterone serves a variety of other functions, including helping regulate immune responses [83], fostering foetal development [84] and counteracting the effects of oestrogen [85]. It follows that, in some contexts, the specific oestrogen-to-progesterone interplay might be more important than the individual levels of these hormones [86, 87]. Together, oestrogen and progesterone are key drivers of reproductive function, and the presence of their receptors in tissues central to athletic functioning, such as skeletal muscle, tendons and ligaments, indicates that that their roles extend beyond reproduction [66, 88].

### Menstrual Cycle Phases

**Fig. 4:**
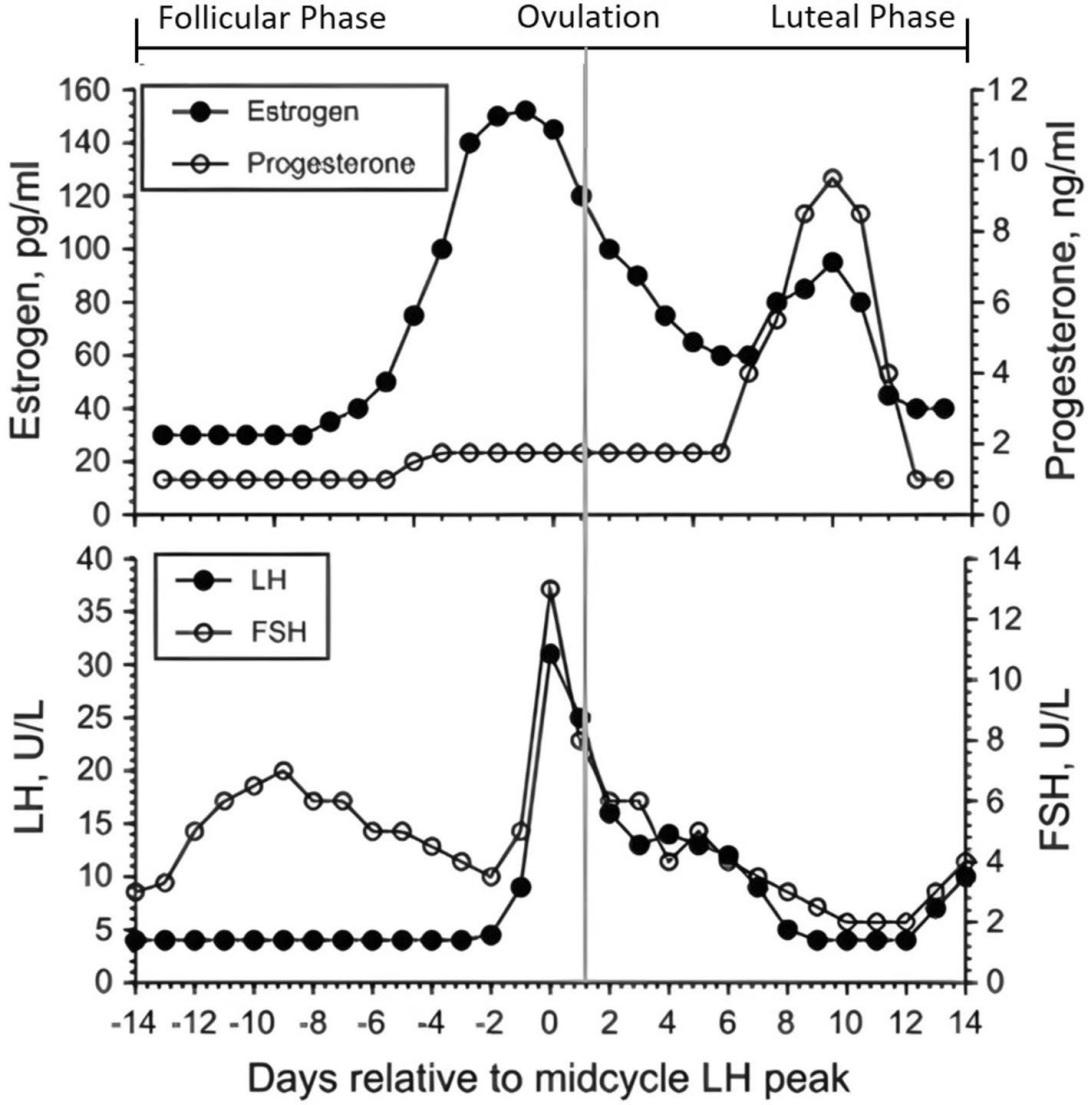
Changes in oestrogen and progesterone (*top*) and luteinising hormone (LH) and follicle-stimulating hormone (FSH) [*bottom*] across the menstrual cycle. Taken from [66] with permission

The menstrual cycle encompasses an intricately orchestrated sequence of biological events, including the ovarian and endometrial cycles (Table 1) [34]. Typically, the menstrual cycle is divided into two main phases: the follicular phase, which begins on the first day of menstruation and continues until ovulation, and the luteal phase, which follows ovulation. Because of substantial fluctuations in oestrogen and progesterone levels across these two phases (Fig. 4), researchers often divide the menstrual cycle into additional sub-phases for greater specificity, with different approaches being adopted across the literature [22, 89–91].

### Menstrual Tracking in Sports

Given the large intra-individual and inter-individual variability in hormonal profiles and cycle timelines throughout the menstrual cycle [92], menstrual patterns often deviate from the idealised representation shown in Fig. 4. Consequently, accurately tracking menstrual cycle phases in sport is a challenging endeavour. The follicular and luteal phases are separated by menstrual bleeding and ovulation. The onset of menstrual bleeding typically marks the start of the follicular phase and the end of the luteal phase. This phase transition is relatively straightforward to identify using self-reported bleeding patterns, a method widely adopted in applied sports contexts because of its simplicity [93]. However, sociocultural factors, such as discomfort in discussing menstrual health with male support staff [64], can present some obstacles for this method.

Identifying ovulation, which marks the transition to the luteal phase from the follicular phase, is considerably more complex. Transvaginal ultrasound remains the gold standard for confirming ovulation by directly observing follicle development and egg release [94]. However, its high cost, reliance on specialised equipment, the invasive nature of the testing and the requirement for trained personnel make it impractical in most sports settings. More accessible alternatives include urinary ovulation kits [93], which detect the luteinising hormone surge occurring 9–26 h before ovulation [41, 91, 95]. Another method involves tracking changes in cervical mucus, which takes on a ‘raw egg white’ consistency around ovulation [94, 96]. However, this method requires a thorough education to ensure accurate recognition of mucus patterns. Basal body temperature (BBT) tracking is another popular method, relying on a typical post-ovulation temperature rise of ~ 0.3 °C that persists during the luteal phase [41]. However, basal body temperature is highly sensitive to external factors such as stress, illness (e.g. fever), disrupted sleep and general measurement errors, which can compromise its reliability [97]. Furthermore, some ovulatory cycles do not exhibit the expected temperature changes, further limiting its accuracy [98].

Blood tests offer the most detailed insights into menstrual cycle phases, particularly for identifying subphases such as the mid-luteal phase, characterised by elevated oestrogen and progesterone levels, and for confirming eumenorrheic cycles (Table 1) [22, 99, 100]. A single progesterone measurement of > 16 nmol∙l-1 is commonly used to confirm an eumenorrheic cycle; however, because of the pulsatile nature of progesterone secretion, more regular sampling throughout the luteal phase provides a more accurate assessment [101]. While blood sampling supports phase identification, it does not capture inter-individual variability in sensitivity to hormonal changes [102], an important consideration in this area of research. Furthermore, because of the invasive nature and resource requirements of routine blood monitoring, regular blood testing is considered by some to be an impractical ideal in certain contexts [103, 104], but not impossible [101]. While emerging alternatives, such as saliva and urinary hormone testing, may offer less invasive and more scalable options for the future when blood tests are not possible or unethical [103], these approaches also do not address differences in sensitivity to hormonal changes and their accuracy is yet to be determined.

The simplest and most widely used method in sports settings remains calendar-based counting, which makes assumptions about menstrual cycle phases based on the first day of menstruation. While this method is convenient and may provide some insight into cycle length and symptom occurrence at specific points during the cycle, it is important to note that it does not accurately identify specific cycle phases, as it cannot confirm ovulation or the post-ovulation rise in progesterone [100, 105]. Additionally, the prevalence of luteal phase-deficient or anovulatory cycles (Table 1) is not well documented in athletes, but has been reported to be as high as 48%, with up to 79% of cycles affected [106]. Consequently, the calendar-based method fails to account for these occurrences [107].

For a more comprehensive review of the available methods for verifying menstrual cycle phases and their limitations, the following articles are recommended [22, 41]. For insights on the ethical risks associated with menstrual tracking in sports, readers are referred to the following texts [108, 109].

### Reproductive Hormonal Profiles and Individual Responses Across the Life Span

In female individuals, fluctuations in reproductive hormones are not limited to the reproductive years but occur across the entire life span, evolving through distinct physiological stages. These stages, which range from prepuberty to post-menopause and outlined in Table 2, reflect diverse hormonal profiles and biological changes that hold critical implications for health, performance and menstrual management strategies [110].

**Table 2:** Stages of reproductive hormone profiles in female individuals across the life cycle. Adapted from McNulty et al. [110]

Stage	Definition
Prepuberty	The stage before peri-puberty, characterised by hormonal dormancy and immature reproductive function
Peri-puberty	The phase before the onset of menstruation, when the young female body begins to undergo changes (e.g. development of secondary sex characteristics)
Puberty	The onset of menarche, marked by significant hormonal changes and the development of reproductive capability
Reproductive years	The phase of optimal fertility in female individuals, during which they may experience various hormonal profiles, such as regular or irregular menstrual cycles, usage of hormonal contraceptives, pregnancy and the postpartum period
Perimenopause	The transitional phase preceding menopause, characterised by hormonal fluctuations and irregular menstrual cycles. Menopause being defined as the permanent cessation of menstruation resulting from the loss of ovarian follicular activity [111]
Post-menopause	The stage following menopause, marked by stabilised low levels of oestrogen and progesterone

To further address the diversity across these menstrual life stages, the framework categorises hormonal milieus into four distinct groups, outlined in Table 3: typical unaltered, typical altered, atypical unproblematic and atypical problematic. These categories serve as markers along a continuum of menstrual-related functioning that ranges from normative to pathological.

**Table 3:** Classification of reproductive hormonal profiles across the life span

Typical unaltered	This category describes natural hormonal and biological functioning that aligns with population norms across life stages, from prepuberty to post-menopause
Typical altered	Refers to individuals with altered hormonal profiles due to widely adopted interventions acting on the menstrual cycle, such as hormonal contraceptives. These are prevalent among athletes and therefore not considered deviations from the norm [112]
Atypical unproblematic	This includes deviations from normative ovulatory and hormonal profiles that do not negatively affect individuals beyond standard menstrual variations. Examples include hormonal changes during in vitro fertilisation [110], the use of hormone replacement therapy [113] or sporadic luteal phase-deficient or anovulatory cycles [114]
Atypical problematic (dysfunctional)	Encompasses abnormal menstrual functioning, hormonal imbalances, and gynaecological or endocrinological disorders that lead to significant short-term or long-term negative health impacts. Common examples may include frequent anovulatory or luteal phase-deficient cycles [115], oligomenorrhoea [40], polycystic ovary syndrome [44], polymenorrhoea [30] and secondary amenorrhoea [48] (Table 1)

While menstrual phases and reproductive stages are commonly defined using characteristic hormonal profiles, it is important to acknowledge that circulating hormone levels alone *may not possess sufficient explanatory power* to reliably predict physiological responses to hormonal changes *in certain contexts*. Inter-individual variability in hormone-receptor sensitivity represents a key mechanism explaining why women with similar circulating hormone levels may experience different outcomes [116]. At the molecular level, oestrogen and progesterone exert their effects through multiple receptor subtypes, whose expression varies across tissues and influence downstream signalling pathways [117]. Studies in human tissues provide complementary evidence, demonstrating inter-individual differences in responses to the same hormone exposure, independent of baseline receptor expression levels [118]. These variations are likely influenced by factors such as differences in intracellular signalling, tissue microenvironment and epigenetic modifications [118]. While this area remains under-researched owing to the complexity of measuring and interpreting receptor-level differences, recognising this variability is crucial for understanding the heterogenous effects of sex hormones and for designing studies that account for individual differences in physiological outcomes.

## Available Interventions

As the symptoms and effects associated with the menstrual cycle constitute the primary mechanisms through which menstrual health may influence various sports-related outcomes [119], interventions acting on these symptoms and effects are of the utmost importance. Within the proposed framework, interventions are broadly classified into biologically acting and non-biologically acting (Table 4).

**Table 4:** Examples of biologically acting and non-biologically acting menstrual-related interventions

Category	Intervention type	Examples	Function
Biologically acting	Pharmacological	Antipyretic medication (e.g. non-steroidal anti-inflammatory drugs and acetaminophen, such as paracetamol), antifibrinolytic agents (e.g. tranexamic acid) [128]	Reduce pain and inflammation, reduce heavy menstrual bleeding
	Pharmacological	Hormonal contraceptives [112]	Contraception, reduce/stop menstrual flow, alleviate symptoms
	Pharmacological	Antidepressants (e.g. selective serotonin reuptake inhibitors) [129]	Alleviate severe mood-related PMS/PMDD symptoms
Biologically acting	Non-pharmacological	Exercise [121]	Reduction of symptoms through release of endorphins and anti-inflammatory effects of exercise
	Non-pharmacological	Dietary adjustments (e.g. vitamin D, calcium, magnesium, zinc and curcumin) [122]	Reduce inflammation, pain modulation and address potentially altered macronutrient needs at specific times during the cycle
	Non-pharmacological	Stress management techniques (e.g. yoga, meditation) [123]	Alleviate menstrual pain through stress management
	Non-pharmacological	Heat therapy [130]	Relief of menstrual cramps through muscle relaxation; enhance pelvic blood flow to reduce fluid retention and swelling
	Non-pharmacological	Sleep management [131]	Address the increased need for sleep during certain phases of the menstrual cycle
Non-biologically acting	Hygiene products	Sanitary pads, tampons, menstrual cups, leakproof underwear [124]	Manage menstrual flow; maintain hygiene; reduction of school, work, and sport absenteeism
Non-biologically acting	Education	Understanding how to manage symptoms and when to seek help [125]	Improved recognition and management of menstrual cycle symptoms

*PMDD* premenstrual dysphoric disorder, *PMS* premenstrual syndrome.

Biologically acting interventions are those that target biological systems and physiological processes to modify the menstrual cycle and its associated symptoms. Typically, these are pharmacological in nature, with some common examples including hormonal contraceptives that alter hormonal levels [120], and medications that help manage menstrual symptoms such as pain (e.g. non-steroidal anti-inflammatory drugs) or heavy menstrual bleeding (Table 1) [e.g. tranexamic acid] [121]. Non-pharmacological interventions that are biologically acting include lifestyle modifications such as regular exercise [121], dietary adjustments [122] and stress management techniques [123].

While the distinction between biologically and non-biologically acting interventions is not always clear-cut, non-biologically acting interventions are typically aimed at managing the symptoms and potential negative effects of the menstrual cycle without directly acting on biological systems. Common examples include access to hygiene products [124], menstrual management education [125] and adapting training regimes in sports [126]. Ultimately, the accessibility and utilisation of a particular intervention for an athlete can be influenced by many contextual and environmental factors, with some common examples including geographic location and socioeconomic status [60], cultural and religious background [13], and menstrual health literacy [127].

## Menstrual-Related Symptoms and Effects, and Their Influence on Relevant Sports-Related Outcomes of Interest

The menstrual cycle, its physiological processes, and relevant (biologically and non-biologically acting) interventions, have been associated with various symptoms and effects that may influence key sporting outcomes of interest. These effects are predominantly attributed to hormonal fluctuations throughout the cycle, which are theorised to impact multiple psycho-physiological systems relevant to athletes [3]. It follows that, menstrual-related symptoms and physiological, psychological and cognitive changes are widely believed to affect various sporting outcomes of interest such as training and performance, health (e.g. injury, illness and psychological well-being), and socio-behavioural or participation outcomes. The following sections provide a brief introduction into the most prominent theories and hypotheses regarding the impact of menstrual health on these outcomes. However, a deep dive into the various theories, hypotheses and supporting levels of evidence is outside the scope of this work, and references are provided to guide readers seeking more detailed discussions.

### Sports Training and Performance Outcomes

Understanding the relationship between the menstrual cycle and sports performance is a topic of growing interest in the applied sporting world. Research in this area primarily investigates how menstrual cycle symptoms (e.g. cramps, heavy bleeding) and hormonal fluctuations may affect athletic outcomes [3]. These symptoms and hormone-driven changes in psycho-physiological functioning are hypothesised to influence critical athletic measures such as strength, power, speed, endurance, recovery, cognition and overall competitiveness [3]. However, evidence across most of these domains remains limited and inconsistent. Many studies are constrained by inadequate sample sizes, suboptimal methodological designs and insufficient verification of menstrual cycle phases, which makes it difficult to draw firm conclusions [132]. Additionally, substantial variability in both hormonal profiles and symptom expression likely contributes to the inconsistent findings reported in the literature [133]. For readers seeking more detailed overviews of studies examining the impact of the menstrual cycle on sports performance, several detailed reviews are available [132, 134–136].

#### Athlete Perceptions and Notable Symptoms

Athletes widely perceive the menstrual cycle as influencing their sports performance [134], with symptoms such as cramps, fatigue and mood swings commonly reported [119]. These symptoms are prevalent in the days leading up to or during menstruation, periods often viewed as the least desirable to compete [137]. For instance, one study found that 93% of athletes reported experiencing menstrual-related symptoms and 67% believed these symptoms impaired their performance [128], although such symptoms rarely lead to missed training sessions [120]. Importantly, perceived impacts of the menstrual cycle on performance vary significantly across individuals [119]. Moreover, discrepancies often emerge between subjective reports and objective performance measures, suggesting that psychological perceptions, potentially driven by placebo effects, can shape, amplify or diverge from physiological outcomes [134]. Thus, while athlete experiences are valuable, their interpretation may be strengthened when considered alongside objective physiological and performance-outcome data.

#### Muscle Activation, Force Production and Power Output

Hormonal fluctuations across the menstrual cycle, particularly variations in oestrogen and progesterone, are theorised to influence muscle activation, force production and power output, primarily through neurological mechanisms [138]. For example, elevated oestrogen levels during the late follicular phase may enhance cortical excitability and motor unit firing rates [139, 140], potentially boosting strength, while progesterone may have inhibitory effects [141]. Some studies report increased strength during high-oestrogen phases [142–145], but findings remain inconsistent, partly owing to methodological differences and variation in muscle groups tested [136, 146, 147]. Beyond neurological influences, oestrogen may counteract the reduction in muscle performance induced by phosphate accumulation during isometric tasks [142]. In contrast, dynamic and power-based activities, such as sprinting, appear to be largely unaffected. This is likely because of the lower sensitivity of type II fibres to hormonal shifts [148, 149]. Overall, while the mechanistic pathways discussed above are plausible, most studies report no significant changes in strength or power across the menstrual cycle, highlighting the need for more nuanced and precise research designs.

#### Endurance Outcomes

Endurance performance may be influenced by menstrual cycle-related hormonal fluctuations through effects on substrate metabolism [133], thermoregulation [98, 150] and cardiorespiratory function [151, 152], though evidence remains controversial and inconclusive [92, 133, 153]. Elevated oestrogen is theorised to enhance fat oxidation and glycogen sparing during submaximal exercise [154–156], while progesterone may counteract these effects [157, 158]. Hormonal fluctuations can also alter cardiovascular and respiratory responses, such as increased heart rate [159–162] and enhanced ventilation [163–168] during the luteal phase. However, other studies have reported no significant alterations in these responses [169–176], and environmental conditions may further modulate cardiorespiratory outcomes [177]. Additional factors such as mood changes [178, 179], sleep disturbances [180] and recovery disruptions during certain phases [180, 181] may further affect endurance capacity. Despite various proposed mechanisms, most studies report no consistent or significant changes in endurance performance across the menstrual cycle [164, 168, 171, 174, 177, 182].

### Athlete Health Outcomes

The menstrual cycle is increasingly recognised as a critical marker of overall health, with some experts proposing it as a fifth vital sign [1]. Alongside performance, the effects of the menstrual cycle on the health and well-being of athletes are a prominent topic in sports science and medicine. This is underscored by a recent article from the International Olympic Committee, which identified “menstrual and other gynaecological health” as the first of ten domains of female health [183]. Menstrual irregularities, such as amenorrhoea, are associated with numerous adverse health outcomes. These include compromised bone health [184], increased risk of athletic injuries [185], altered immune responses [186], increased risk of cardiovascular disease [187] and negative mental health effects [37]. These outcomes can further influence training and performance, underpinning the importance of a regular menstrual cycle as an indicator of overall health [188].

#### Illness Outcomes

Menstrual-related illness in athletes, though underexplored, can result in time-loss [189] and long-term health consequences [190]. This includes menstrual cycle disturbances such as functional hypothalamic amenorrhoea (Table 1), anovulatory cycles and luteal phase deficiencies [191], which may arise from low energy availability or pre-existing vulnerabilities [107]. While some athletes may perceive amenorrhoea as beneficial because of the absence of bleeding, it carries significant health risks, including reduced bone density [184], cardiovascular issues [192] and increased susceptibility to mood disorders [37]. Other more subtle disturbances often go undetected because of unchanged bleeding patterns, and their potential effects on health and performance remain underexplored [58, 193]. In addition, reproductive health disorders such as endometriosis and PCOS (Table 1) can significantly impair well-being and training capacity, with conditions like endometriosis frequently undiagnosed because of the normalisation of menstrual pain [194].

Exercise plays a dual role in menstrual health. Moderate physical activity can alleviate menstrual symptoms [195, 196], improve ovulation in women with PCOS [73] and contribute positively to reproductive health [197]. However, excessive exercise, especially in the context of low energy availability, may lead to menstrual dysfunction and other adverse outcomes, as described within the Relative Energy Deficiency in Sport (REDs) framework (Table 1) [47, 198, 199]. However, despite its growing clinical use, it is important to note that REDs has faced recent criticism for its lack of validated biomarkers, limited mechanistic specificity and ongoing uncertainty around its causal structure [200]. Critics argue that REDs describes a cluster of non-specific symptoms — such as menstrual dysfunction and performance decline — that overlap with other physiological conditions, raising concerns that it may oversimplify complex biological processes and remain in need of stronger empirical validation as a coherent clinical construct [200].

#### Injury Outcomes

The injury outcomes component of the framework predominantly considers how hormonal fluctuations across the menstrual cycle may influence injury risk through effects on musculoskeletal tissues, while also incorporating other relevant pathways and contributing factors. Oestrogen, in particular, has a dual role, supporting bone and muscle function while reducing tendon and ligament stiffness, which may contribute to varying injury susceptibility across the cycle [201]. This may be especially relevant to anterior cruciate ligament (ACL) injuries, which occur 1.5–3.6 times more frequently in female athletes [202–205] and are predominantly non-contact in nature [206]. Proposed mechanisms include hormonal influences on collagen synthesis and ligament laxity, with elevated oestrogen levels potentially compromising knee stability [207, 208]. However, evidence linking specific menstrual phases to an increased ACL injury risk is inconsistent, with different studies identifying risk at different points across the cycle [209–215].

Beyond ACL injuries, findings on muscle and tendon injuries are inconsistent. One study reported increased muscle injury incidence in the late luteal phase [8], potentially linked to hormone withdrawal, adverse symptoms (e.g. fatigue or lower back pain),and inflammation [216], while other studies associate a higher risk with the late follicular phase [7, 217]. Discrepancies likely arise from variations in study design [8], phase classification methods [218], sample characteristics [8], as well as from inappropriate analytical practices, including omitting inconvenient data points or p-hacking of data [219–221]. Additionally, athletes with atypical hormonal profiles, due to menstrual irregularities or hormonal contraceptive use, may have different injury risks. Amenorrhoea, for instance, has been associated with increased musculoskeletal injuries [222], and contraceptive use may affect factors such as bone density [223], connective tissue [224] and muscle properties [225], although findings remain inconclusive [208, 226], particularly for non-oral contraceptives [185]. These complexities underscore the need for more precise, hormone-confirmed studies to clarify any contributions to injury risk patterns.

#### Psychological Outcomes

The psychological health of female athletes is shaped by a unique intersection of athletic demands and reproductive health challenges. Some evidence suggests that female athletes are at greater risk for anxiety and depressive disorders than their male counterparts [227] and non-athlete peers [228, 229]. These risks are heightened during certain menstrual phases and reproductive transitions such as pregnancy, postpartum and menopause [66, 230–232]. Examining the relationship between menstrual health and psychological outcome is important, as it can affect athletes’ well-being and performance. This section introduces two primary aspects of psychological-related outcomes: (1) the impact of menstrual health on athletes’ psychological health and (2) the potential of physical activity and sports participation to support or challenge psychological health.

Hormonal fluctuations, particularly declines in oestrogen and progesterone during the late luteal phase, can trigger mood disturbances, irritability and feelings of low energy [230, 231, 233, 234]. These effects are compounded by physical symptoms (e.g. cramping, bloating) [53, 235], body image concerns [236, 237] and menstruation-related stigma [238, 239]. Menstrual disorders such as premenstrual syndrome and premenstrual dysphoric disorder (Table 1) further intensify these psychological effects, with premenstrual dysphoric disorder linked to severe emotional distress [240–242]. Additionally, the psychological impacts of reproductive conditions such as endometriosis and PCOS in athletes remain poorly understood, pointing to key research gaps.

Despite these challenges, physical activity and sport participation can help mitigate menstrual-related psychological symptoms. Exercise has been shown to support mood regulation by increasing dopamine and serotonin levels [243], and improving sleep [244], body composition [245] and physical symptoms [121]. Moreover, the social environment in sport, through support from teammates, coaches and peers, provides an ideal setting to reduce stigma, create a supportive environment, and enhance emotional well-being [246], while also offering valuable opportunities for menstrual health education to help athletes better manage emotional challenges [127]. Together, these benefits position sport as both a psychological buffer and an empowering space for female athletes navigating menstrual health. Nevertheless, it is important to recognise that competitive sports environments can also contribute to negative psychological health outcomes, and in some sporting contexts, the menstrual cycle remains a taboo topic, which can limit open communication [238].

### Sports Participation and Socio-Behavioural Outcomes

Physical exercise and sports are well established as essential contributors to improved physical health [247]. However, the benefits of regular physical activity often extend far beyond physical well-being, fostering a range of socio-behavioural outcomes such as a sense of belonging [248], opportunities for social connection [249], teamwork development [250] and enhanced self-efficacy [251]. Despite these benefits, women drop out of sports at higher rates than their male counterparts, with factors such as negative experiences and stigma surrounding the menstrual cycle emerging as significant barriers [64, 252].

Alarmingly, data from the Youth Sport Trust in England revealed that among 6653 surveyed girls, 39% reported their periods as a barrier to sports participation [253]. Menstrual symptoms, including cramps and heavy bleeding, can hinder performance and participation because of discomfort, pain or fear of leakage [254, 255]. Beyond the physical challenges, social factors also contribute. Negative experiences in sports, such as coaches dismissing menstrual-related symptoms as weakness or excuses, combined with pervasive stigma surrounding menstruation, exacerbate disengagement [15, 64]. Anxieties about how menstruation is perceived by others, coupled with inadequate support from coaches and teammates, have been identified as significant barriers to physical activity [256]. These challenges are further compounded by the lack of open discussions about menstrual health, which often leads to feelings of isolation and discomfort [9].

To address these concerns, improvements in menstrual health literacy among coaches and athletes is needed to foster a more supportive and inclusive environment that acknowledges and accommodates menstrual health needs [127, 257]. Such efforts can help to keep female individuals involved in physical activity, ensuring that female individuals feel empowered regardless of their menstrual cycle experiences [110]. However, menstrual health is only one factor contributing to the higher dropout rates among female athletes compared with male athletes. Other contributing factors include competing interests, social influences and financial constraints [258, 259].

## Beyond Exploratory Research: Causal Inference, Validation and Intervention

The framework presented in this article is intended as a broad heuristic structure that organises key concepts in menstrual health in sport, supports theory-driven inquiry and encourages greater attention to underlying mechanistic pathways. By design, however, the framework is informal and conceptual; it is not itself a statistical model and is therefore not suitable for robust statistical analysis, formal causal modelling or validation.

Establishing causal effects, regardless of the study design, requires a well-defined causal question grounded in explicitly specified assumptions about the underlying causal structure. Randomised controlled trials are commonly viewed as a particularly powerful design and often considered the gold standard for estimating causal effects under well-defined conditions [260]; however, the notion that RCTs occupy a privileged or universally superior epistemic status is increasingly contested within the causal inference literature [28, 261, 262]. Regardless, in menstrual health and sport research, the feasibility of RCTs is frequently limited by ethical, logistical and biological considerations. For example, it is not possible to randomly assign hormonal fluctuations across the naturally occurring menstrual cycle, precluding the use of traditional RCTs to investigate causal effects of natural hormonal variation on outcomes such as performance or injury risk. Moreover, in certain contexts, tightly controlled experimental designs involving exogenous manipulation of hormonal levels may be ethically inappropriate or practically infeasible because of the risk of disrupting athletes’ normal physiological and menstrual processes or regulatory prohibitions on performance-enhancing substances (e.g. testosterone). Consequently, research in this domain has tended to rely predominantly on observational or quasi-experimental data, primarily without an explicit and coherent causal framework to guide the study design, analysis and interpretation.

To address these challenges and enable more rigorous validation, the use of causal DAGs is advocated. Situated within the structural causal model framework, DAGs provide formal mathematical representations of hypothesised causal systems, making assumptions explicit and enabling principled reasoning about identification (Table 1), estimation and validation [26–29]. Within this framework, causal effects are defined in counterfactual terms [26–28]—that is, in terms of how an outcome would be expected to differ under a hypothetical intervention that assigns an exposure to an alternative value, relative to what would have occurred otherwise. Framing causal questions in this way clarifies both the nature of the assumed intervention and whether effects are conceptualised as continuous (e.g. dose–response) or discontinuous (e.g. threshold or binary) relationships. Once a counterfactual causal question is specified, the central task becomes determining whether that causal contrast is identifiable from the available data and assumptions.

**Fig. 5:**
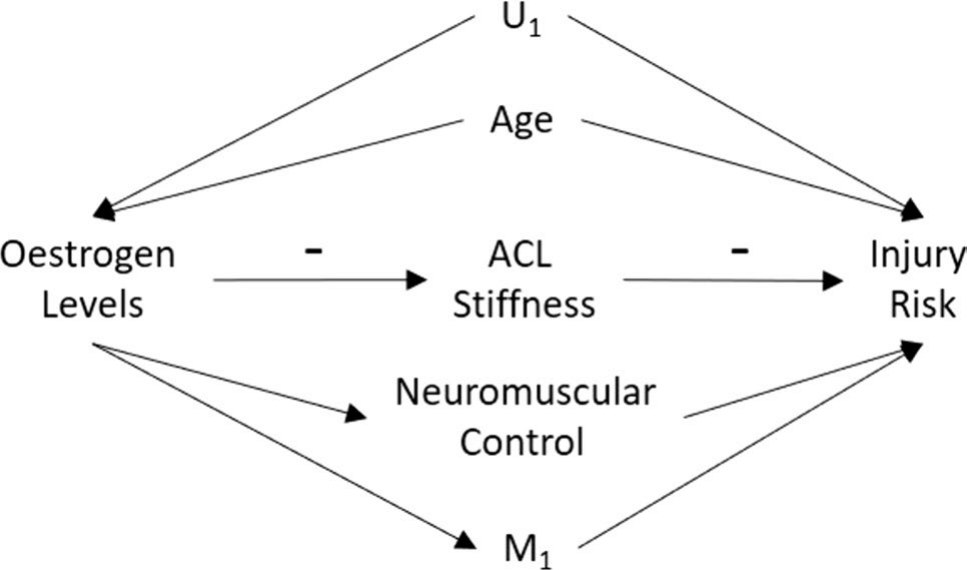
An example of a causal directed acyclic graph illustrating that oestrogen levels are hypothesised to have a causal effect on injury risk, mediated through three identified pathways; anterior cruciate ligament (ACL) stiffness, neuromuscular control and M_1,_ an unmeasured mediator. *Arrows* represent a quantifiable causal effect. (+ / −) symbols are used to represent a positive (increasing) and negative (decreasing) effect. For example, while no ( +) symbols are present, the ( −) symbols illustrate that heightened oestrogen levels cause lower levels of ACL stiffness, and heightened ACL stiffness results in lower injury risk. Age is a confounder (common cause) that biases the relationship between oestrogen levels and injury risk, while U_1_ represents an unmeasured confounder. By testing and demonstrating reproducible relationships between variables as hypothesised by the directed acyclic graph, evidence supporting the validity of the measures (e.g. oestrogen levels, ACL stiffness and injury risk) and the proposed causal framework, i.e. the directed acyclic graph in this instance, is provided. Note: this directed acyclic graph is presented for illustrative purposes and should not be interpreted as a complete example

Directed acyclic graphs support this task through graphical theorems and criteria such as d-separation, the back-door and front-door criteria, together with the do-calculus, which together clarify whether causal effects are identifiable and, if so, which variables must be controlled for to obtain unbiased estimates [24, 26–28]. When the requisite assumptions hold (including conditional exchangeability, positivity, consistency and correct model specification), causal effects can be identified and estimated from observational data [26–29]. Importantly, DAGs are not restricted to observational research: they inform study design, covariate selection, mediation analysis and interpretation across experimental, prospective and observational contexts alike. An illustrative example of a causal DAG is presented in Fig. 5.

As visually represented in the example presented in Fig. 5, it is hypothesised that fluctuations in oestrogen (exposure) influence ACL injury risk (outcome) through three distinct mediating pathways: (1) alterations in ACL stiffness; (2) neuromuscular control; and (3) an unmeasured mediator, denoted M_1_. The causal DAG also incorporates two confounders that bias the relationship between oestrogen levels and ACL injury risk—age and an unmeasured confounder, denoted U_1_. This explicitly defined causal model offers a testable mathematical model for evaluating causal relationships, exploring diverse pathways, conducting mechanistic studies, and implementing more rigorous validation processes, paving the way for new research avenues beyond basic exposure–outcome relationships.

### New Research Avenues, Causal Mediation and Sensitivity Analysis

Shifting beyond basic exposure–outcome associations, a causal model that clearly incorporates proposed mechanistic pathways, such as the link from increased oestrogen levels to diminished ACL stiffness and elevated injury risk, unlocks more nuanced and purposeful research avenues. For example, instead of simply documenting associations between hormonal changes and injury risk, this approach empowers researchers to also investigate the intermediate mechanisms driving causal effects. For example, controlled laboratory studies can test the physiological plausibility of the oestrogen–ACL stiffness connection, while longitudinal field studies can explore whether declines in ACL stiffness cause injury. This multi-tiered strategy strengthens causal inference by aligning empirical investigations with hypothesised mechanisms, laying a foundation for more robust conclusions about the processes at play.

Expanding on this, a major strength of employing causal DAGs lies in their capacity to establish identification and guide the estimation of causal effects transmitted through specific mechanistic pathways [24, 26–28]. To elaborate, rather than estimating only the total effect of an exposure on an outcome, causal DAGs clarify the conditions for *causal mediation analysis* (Table 1), which, under appropriate assumptions, decomposes the total causal effect into distinct direct and indirect pathways. For example, as per the DAG presented in Fig. 5, causal mediation analysis can be used to estimate the relative contributions of each mediating pathway of oestrogen’s effect on ACL injury risk. This decomposition clarifies the role of underlying mechanisms in shaping the outcome and can help identify potential intervention targets.

However, this approach hinges on certain assumptions, including the absence of unmeasured confounding among exposure, mediators and outcome. While the confounding produced by age can be managed by controlling for that variable, the presence of U_1_ as an unmeasured confounder is problematic, biasing the estimates of the effect of oestrogen on injury risk. In scenarios such as these, a sensitivity analysis (Table 1) can be applied [26–28]. This method evaluates the robustness of estimated effects under varying assumptions about unmeasured confounding, mediation misspecification or measurement error. For instance, a sensitivity analysis can estimate the strength of U_1_’s influence required to attenuate or nullify the effect of oestrogen via the three identified causal pathways or explore the impact of measurement error in ACL stiffness or neuromuscular control on mediation estimates.

As noted, causal DAGs can strengthen the design and interpretation of observational studies by making causal assumptions explicit and guiding appropriate identification strategies. In some cases, carefully designed observational analyses, including target trial emulation, have produced findings that align with results from RCTs [263]. However, observational causal inference does not guarantee replication in experimental settings, and there are well-documented instances where effects suggested by observational analyses were not confirmed in subsequent RCTs [264–266]. These examples highlight the potential limitations of this approach and underscore the value of triangulating evidence across study designs when drawing causal conclusions in complex systems such as female athlete health.

### Validation, d-separation and Conditional Independence

**Fig. 6:**
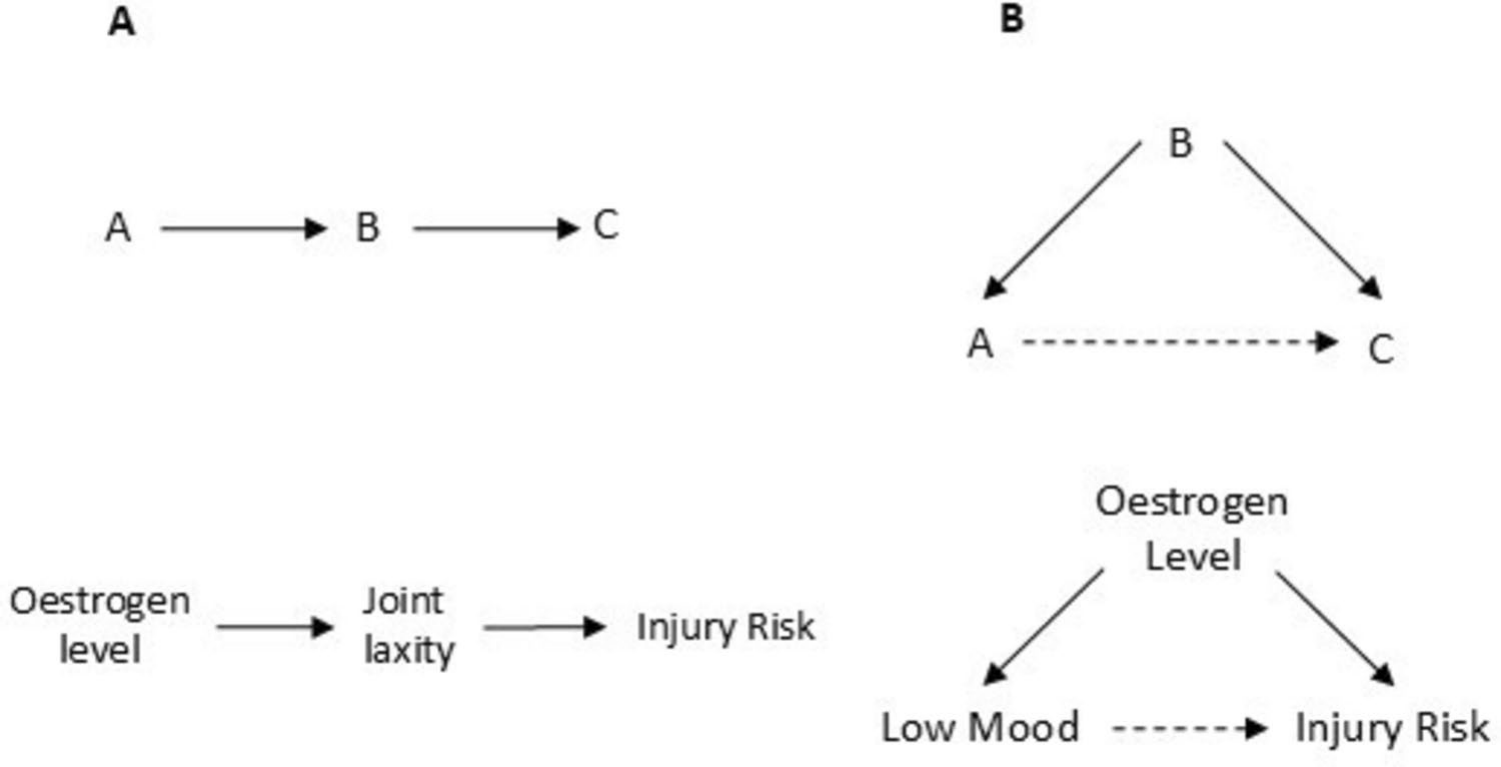
Two causal directed acyclic graphs illustrating the fundamental structures of (**A**) mediation and (**B**) confounding. *Solid arrows* represent a quantifiable causal effect. *Dashed arrows* represent spurious (non-causal) associations. In (**A**), the causal effect of A (oestrogen level) on C (injury risk) is hypothesised to be fully transmitted through a single mechanism, B (joint laxity). If the directed acyclic graph provides an accurate reflection of reality, by controlling for joint laxity, oestrogen level and injury risk should become statistically independent (d-separated), indicating that the mediator accounts for the entire effect. In (**B**), because of a confounder (oestrogen level), low mood and injury risk are statistically associated despite lacking a causal relationship, i.e. low mood and injury risk are non-causally (spuriously) related, as represented by the *dashed arrow*. Consequently, controlling for oestrogen level should render low mood and injury risk statistically independent (d-separated), offering a means to validate the confounding structure. If d-separation does not occur in either scenario, it suggests the presence of additional unaccounted variables or pathways, challenging the validity of the proposed models

Building on this integrated research framework, more robust validation methods can strengthen the model’s integrity. A central technique in this process is d-separation (Table 1), a graphical criterion that evaluates the conditional independencies implied and predicted by a causal DAG [26–28]. To illustrate this approach, consider the simplified DAGs in Fig. 6.

In Fig. 6A, where the causal effect of A on C is fully mediated through B, conditioning on B should render A and C statistically independent. By contrast, in Fig. 6B, A and C are statistically associated because of the confounder B, despite no direct causal link between them; conditioning on B should again render A and C statistically independent. These predicted independencies can be empirically tested against observed data, establishing a clear link between mechanistic insights and the model’s validity. Notably, any discrepancies between expected and observed independencies signal potential misspecification, guiding targeted refinements to the model. By integrating mechanistic evidence from controlled experiments and longitudinal studies with rigorous validation tools like d-separation, researchers can develop causal models that are both theoretically robust and practically valuable, offering actionable insights for interventions in sports science and medicine [26–28].

**Fig. 7:**
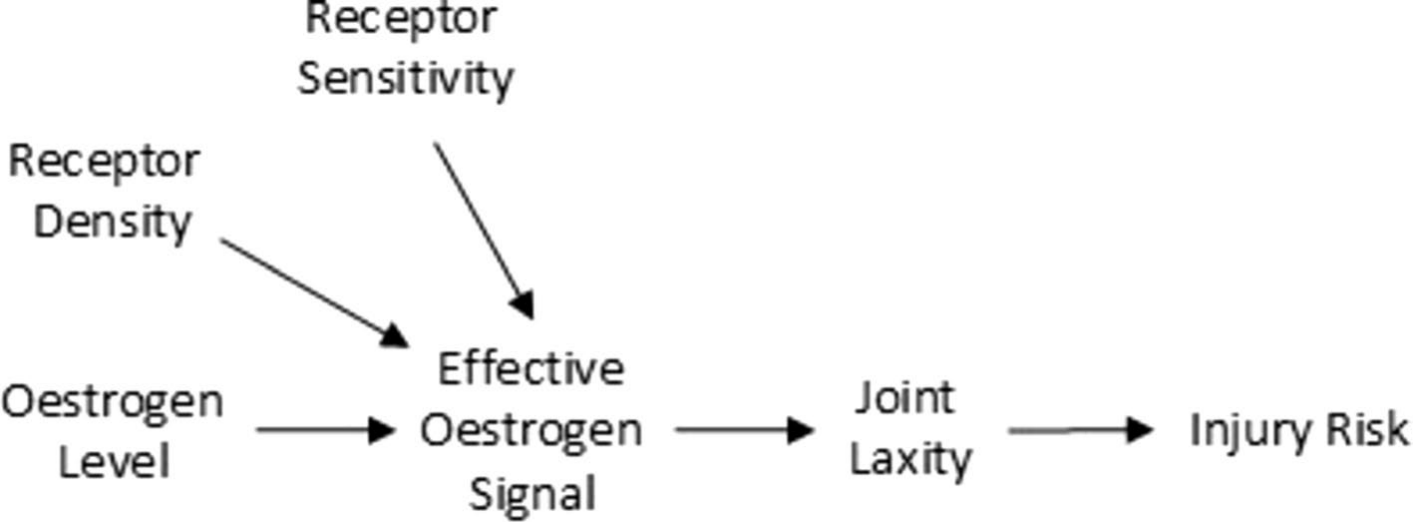
Example of a causal directed acyclic graph illustrating how causal representations may differ depending on the level of biological detail and mechanistic specification. This figure presents a refined causal directed acyclic graph in which circulating oestrogen concentration and receptor-related factors (e.g. receptor density and sensitivity) jointly determine an intermediate construct representing a biologically effective oestrogen signal, which in turn influences joint laxity and injury risk. This representation illustrates how incorporating more proximal mechanistic variables may be necessary when simpler models fail to satisfy predicted conditional independencies or adequately capture biological reality

Importantly, failure of predicted conditional independencies does not merely indicate statistical noise but may reflect an incomplete or overly coarse representation of the underlying causal system. In such cases, refinement of the causal model may be required by incorporating more proximal or mechanistically relevant variables. For example, representing oestrogen solely by the circulating level may be insufficient if downstream biological effects depend on factors such as receptor density, receptor sensitivity or tissue-specific responsiveness [116]. As illustrated in Fig. 7, a more precise causal representation may therefore require modelling the oestrogen level jointly with receptor-related variables, which together determine the biologically effective oestrogen signal. Incorporating such proximal variables can resolve apparent violations of d-separation, improve causal coherence and better align the DAG with known physiological mechanisms, thereby strengthening both identification and interpretation.

Finally, it is important to reiterate that science and validation are inherently iterative processes. Discrepancies between observed data and a framework or model’s predictions can reveal potential issues with the validity of the measures (i.e. their ability to accurately capture the intended constructs) or the causal model itself (e.g. flawed assumptions, misidentified mechanisms or inadequate control of confounding variables). While the examples presented here are intentionally simplistic for illustrative purposes, more comprehensive DAGs could incorporate additional variables, including other relevant confounders, to better represent the complexity of real-world systems and enhance the robustness of validation efforts.

### Levels of Abstraction, Proxy Variables and Causal Interpretation

An important consideration when applying causal inference methods in menstrual health research is the level of abstraction at which variables are defined and modelled. As discussed in Sect. 2, causal DAGs must be constructed at a coherent level of description aligned with the specific causal question being asked. Distinct DAGs may therefore be appropriate for the same substantive phenomenon when questions are posed at different levels of abstraction.

**Fig. 8:**
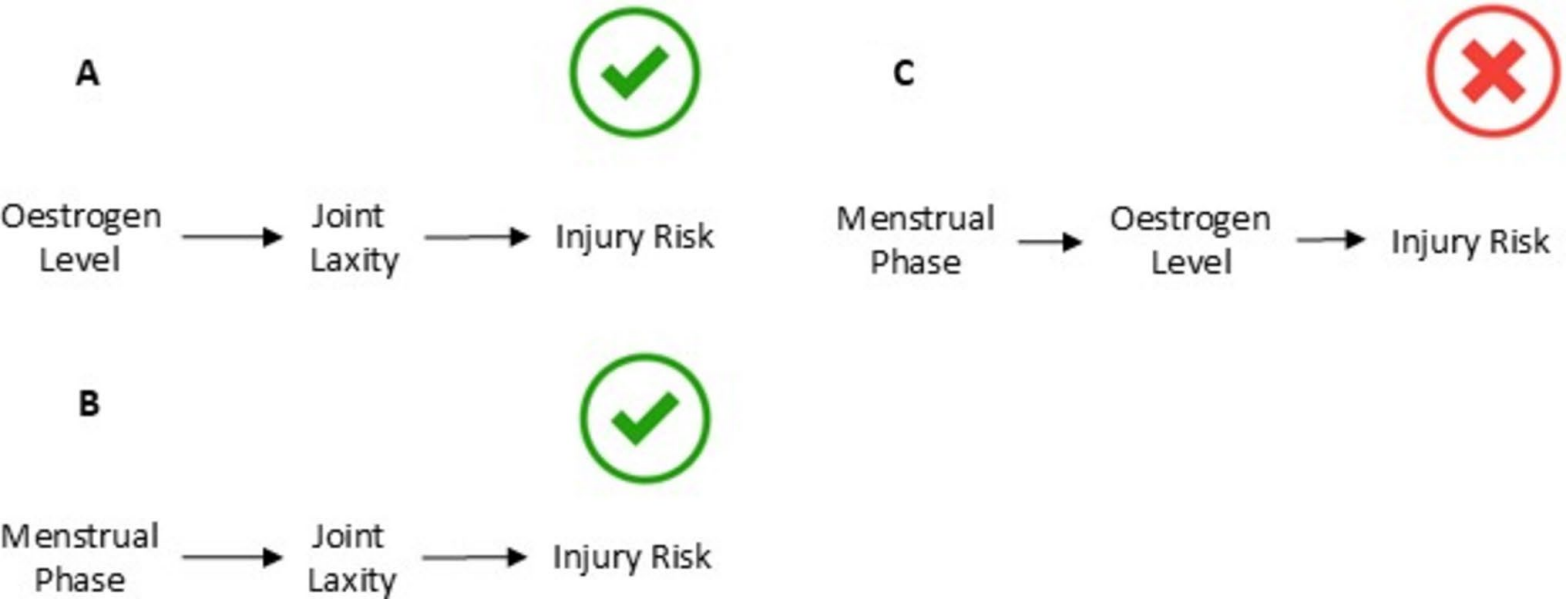
Illustration of causal directed acyclic graphs (DAGs) constructed at different levels of abstraction, highlighting valid and invalid causal representations. (**A**) and (**B**) present two conceptually valid causal DAGs at different levels of abstraction addressing distinct but legitimate causal questions. In (**A**), the oestrogen level is modelled as the exposure, influencing injury risk through the mediator joint laxity, representing a mechanistic hormone-level causal model. In (**B**), the menstrual cycle phase is modelled as the exposure influencing joint laxity and injury risk, representing a higher-level descriptive causal model in which phase may, in principle, be interpreted as a pragmatic exposure or proxy variable when hormonal data are unavailable. Although these DAGs operate at different levels of abstraction, each represents a coherent causal question when considered independently. (**C**) illustrates an invalid causal representation in which the menstrual cycle phase and oestrogen level are included within the same DAG. Because the menstrual cycle phase is a descriptive classification defined by underlying hormonal dynamics, treating phase and oestrogen as causal peers violates causal coherence by modelling a variable alongside its own abstraction. The *red cross* (X) indicates that this structure is not causally admissible, as once hormonal states are specified, the phase does not constitute an independent causal variable and does not admit a meaningful counterfactual intervention. Including both within the same DAG risks cross-level misinterpretation and invalid causal inference

For example, one causal DAG may be specified at a mechanistic level, in which hormone levels such as oestrogen serve as the exposure, physiological properties such as ligament stiffness, joint laxity or neuromuscular control act as mediators, and injury risk is the outcome, as illustrated in Figs. 5, 6, and 7. In contrast, a separate causal DAG may be specified at a higher descriptive level, for example, where the menstrual cycle phase is treated as the exposure influencing downstream physiological mechanisms and outcomes (Fig. 8). In this formulation, the cycle phase may be interpreted causally either as a pragmatic exposure in its own right or as a proxy variable intended to capture systematic variation in underlying hormonal dynamics when direct hormone measurements are unavailable.

While, in principle, and for the purposes of the example presented in Fig. 8, treating the menstrual cycle phase as a proxy for a hormonal state is admissible within a causal DAG framework, substantial inter-individual and intra-individual variability in hormonal profiles across nominal phases raises serious concerns about the validity of the menstrual cycle phase as a reliable proxy for hormone levels [99, 100]. Accordingly, causal interpretations based on phase-level models should be treated with caution and should not be conflated with hormone-specific causal effects.

Crucially, while both formulations presented in Fig. 8A, B are individually coherent, menstrual cycle phase and hormone levels should not be treated as causal peers within the same causal DAG (Fig. 8C). Menstrual phases are higher-level descriptive classifications defined by underlying hormonal trajectories rather than independent biological state variables. Once hormonal states are explicitly specified in a causal model, the cycle phase no longer admits a meaningful counterfactual intervention and therefore does not constitute an independent causal variable. Including both within the same causal DAG would amount to modelling a variable alongside its own abstraction, violating the coherence of the causal representation and risking cross-level misinterpretation.

### A Message of Caution for the Implementation of Causal DAGs

While this article provides a basic introduction into causal DAGs for more robust validation processes in menstrual health research, it is important to emphasise that causal DAGs are complex mathematical tools, and the information presented here is neither comprehensive nor sufficient enough for practical implementation. Despite their apparent simplicity, causal DAGs involve nuances that require deeper understanding to avoid common pitfalls [267], and their applications extend beyond the scope discussed here. The goal of presenting this information here was to introduce menstrual health in sport researchers to causal DAGs, encouraging further study and engagement with these powerful research tools. Researchers are urged to consult additional resources, in-depth literature and experts for accurate application. For further reading into causal DAGs within a sporting context, the following text is suggested [24]. For comprehensive reading on the topic, the following seminal texts are suggested [26–28].

## Conclusions

This article has presented an overarching conceptual framework for menstrual health in sport research, designed to advance the field by systematically organising key constructs—including contextual variables, biological mechanisms, interventions, symptoms, menstrual-related effects, and outcomes such as performance, athlete health, and socio-behavioural functioning—into a coherent system of relationships. The framework should be understood as a flexible conceptual map, useful for integrating diverse domains of menstrual health research and highlighting important avenues for future investigation. While it promotes causal and mechanistic reasoning by encouraging the study of relevant menstrual-related pathways and mechanisms, it remains insufficient for robust causal inference. On its own, the framework cannot provide the formal structure required for robust statistical modelling or empirical validation. Its primary function, therefore, is to provide heuristic value and serve as a starting point for the development of causal DAGs, which make assumptions explicit, enable rigorous analysis and help distinguish correlation from causation. In this way, the framework acts as a starting point to help translate broad conceptual relationships into formalised models capable of testing and refinement.

Ultimately, the framework is best seen as a foundation for a more rigorous causal inference in future research. It organises current knowledge and supports systematic refinement, but its true value will only be realised through the development and testing of causal DAGs that move menstrual health research toward more precise, predictive and evidence-based models.
